# PEDV regulates trans-mammary epithelial migration of T cells in a CCR10/CCL28-dependent manner

**DOI:** 10.1128/jvi.00024-26

**Published:** 2026-03-20

**Authors:** Yuwei Zhai, Yajuan Ma, Chaofan Liu, Yanan Zhang, Feiyan Wang, Qingbo Shi, Qinye Song, Chen Yuan

**Affiliations:** 1 College of Veterinary Medicine, Veterinary Biological Technology Innovation Center of Hebei Province, Hebei Agricultural University 74562, Baoding, China; 2 Hanzhong Animal Disease Prevention and Control Center, Hanzhong, China; Fred Hutchinson Cancer Center Vaccine and Infectious Disease Division, Seattle, Washington, USA

**Keywords:** PED, T cells, CCR10, CCL28, migration

## Abstract

Porcine epidemic diarrhea virus (PEDV) is a re-emerging coronavirus that infects neonatal piglets via newly discovered colostrum transmission routes. Research has shown that T cells carrying PEDV in colostrum can infect piglets, but the mechanism is unclear. This study revealed that PEDV impairs T-cell immune function, preventing the production of granzymes, perforin, and interferon-γ. Furthermore, RNA sequencing of PEDV-infected and uninfected T cells revealed significant differences in gene expression: the expression of CCR10 on T-cell surfaces increased, whereas the expression of vinculin, α-actinin, paxillin, FAK, and talin-1 decreased (*P* < 0.05). The results revealed that CCR10 only interacts with CCL28. PEDV strains led to significant upregulation of CCL28 in porcine mammary epithelial cells (PMECs). Finally, the role of CCR10/CCL28 in T-cell migration during PEDV infection was illustrated via a small interfering RNA (siRNA)-mediated knockdown method and a coculture model of PMECs with T cells. The results demonstrated that the addition of the chemokine CCL28 to the lower chamber of the transwell system elicits a chemotactic effect on T cells. This phenomenon is attributed mainly to the substantial increase in the expression levels of paxillin and FAK on the surface of T cells following siRNA-mediated interference with CCR10. In contrast, no significant differences were observed in the expression levels of vinculin and α-actinin. These results suggest a mechanism underlying the regulation of T-cell migration in response to CCL28, which is potentially associated with the modulation of paxillin and FAK expression levels.

**IMPORTANCE:**

Understanding the impact of porcine epidemic diarrhea virus (PEDV) on T-cell function and migration is crucial. This study reveals PEDV impairs T-cell immunity by preventing key molecule production. It uncovers significant gene expression changes in infected T cells, with CCR10 rising and several adhesion-related proteins falling. Further, CCR10 interacts solely with CCL28, and PEDV upregulates CCL28 in PMECs, is key. The transwell system experiments show CCL28's chemotactic effect on T cells, linked to paxillin and FAK expression changes. These findings shed light on how PEDV manipulates T-cell migration via the CCR10/CCL28 axis, aiding in developing strategies against PEDV.

## INTRODUCTION

Porcine epidemic diarrhea (PED) is an acute and highly contagious intestinal infectious disease characterized by vomiting, diarrhea, dehydration, and death, and is caused by porcine epidemic diarrhea virus (PEDV) (1–3). The disease was first recorded in England in 1971, and PEDV was first isolated in Belgium in 1977 and then spread to other European and Asian countries (4). In 2010, a new highly pathogenic PEDV mutation appeared in China, resulting in a mortality rate of nearly 100% in suckling piglets (5). Since then, PEDV has spread to numerous countries (6). PEDV results in severe economic losses to the swine industry because of its widespread prevalence and high mortality (7).

The main transmission route of PEDV is via the fecal-oral route, usually through contact with vomitus, excrement, and other contaminated items of infected piglets. Other transmission routes include fecal-nasal, aerosol, and semen routes (1, 8, 9). PEDV can be detected not only in sow colostrum but also in colostrum samples from immunized and infected sows (5, 10). Our previous research confirmed that PEDV can be transmitted vertically from sows to neonatal piglets via colostrum. During this process, CD3^+^ T cells play an important role. PEDV colonizing intestinal epithelial cells can be transferred to CD3^+^ T cells in the sow intestine through cell-to-cell contact and then reach the mammary gland through the blood circulation and be transported to the colostrum through mammary epithelial cells, causing infection in piglets (11). However, the mechanism by which virus-carrying T cells migrate to colostrum remains unclear.

In the mammary gland tissue of sows, mammary epithelial cells are closely arranged, forming a continuous layer of alveoli and ducts (12). For the plasmocytes and lymphocytes to reach the sow’s mammary gland and migrate into the alveolar cavities (colostrum), they must undergo deformation movements and pass through the mammary epithelial cells. During these processes, the interaction between chemokine receptors and chemokine ligands facilitates the migration of immune cells within the mammary tissue (13). Chemokine receptors are a type of G protein-coupled receptor with a length of approximately 330–350 amino acids and seven transmembrane regions. On the basis of the specific type of chemokine to which they bind, chemokine receptors can be categorized into four distinct families: CXCR, CCR, CR, and CX3CR. CCR10, a member of the CCR chemokine receptor family, is expressed on the surface of diverse cell types, with a predominant presence on immune cells (14). A comprehensive review of the literature indicates that the expression of CCR10 has a chemotactic influence on the migration of immune cells toward mucosal sites. Specifically, several studies have demonstrated that CCR10 expressed by IgA plasma cells is capable of regulating the migration of these cells to the mammary gland (15).

Furthermore, in a CCR10 knockout model, a significant reduction in the number of IgA antibody-secreting cells that aggregated in mammary gland tissue was observed. This finding indirectly corroborates the crucial role of CCR10 in the migratory process of IgA plasma cells to mammary gland tissue (16). Our previous research revealed that PEDV can modulate the expression of CCR10 on the surface of T cells. Therefore, we speculate that CCR10 may play a crucial role in the migration of T cells across the mammary epithelium.

Here, RNA-seq revealed that the expression of CCR10 on the surface of T cells infected with PEDV significantly increased, whereas the expression of vinculin, α-actin, paxillin, FAK, and Talin-1 significantly decreased (*P* < 0.05). Furthermore, a siRNA-mediated knockdown method and a coculture model of PMECs were used to elucidate the PEDV-mediated CCL28/CCR10 interaction and the downstream expression of paxillin and FAK of CCR10, which recruits T cells to migrate across mammary epithelial cells. Taken together, the results of this work clarify the mechanism by which the interaction between CCL28 secreted by mammary epithelial cells and CCR10 on the surface of T cells facilitates the migration of T cells across mammary epithelial cells, providing unique insights into the vertical transmission mechanism of PEDV.

## MATERIALS AND METHODS

### Cells, viruses, and reagents

PMECs, virulent PEDV QY/2016 strains (GenBank no. MH244927) and CCL28 protein were stored in the laboratory for animal infectious diseases at the College of Veterinary Medicine, Hebei Agricultural University. DMEM/F12 culture medium (catalog no. 12100046), 0.25% trypsin solution (catalog no. 25200114), penicillin-streptomycin antibiotics (catalog no. 15140122), and fetal bovine serum (FBS) (catalog no. A5256701) were obtained from Gibco. Phytohemagglutinin (PHA) (catalog no. 9008-97-3) was purchased from Protech. An enhanced chemiluminescence (ECL) ultrasensitive colorimetric detection kit (catalog no. 36208ES60) and protein marker (10–250 kDa) (catalog no. WJ102) were procured from Yeasen Biotechnology (Shanghai) Co., Ltd. A goat anti-rabbit YF594 (catalog no. Y6107L) was purchased from UE. The mouse anti-CD3e protein conjugated with phycoerythrin (PE) (catalog no. 9515-09) was obtained from South Biotech. A Cell Counting Kit-8 (CCK8) (catalog no. C0037) was obtained from Beyotime Biotechnology Co., Ltd. (Shanghai, China). A Focal Adhesion Protein Antibody Sampler Kit (catalog no. 13430T) was purchased from Cell Signaling Technology Co., Ltd. Cell lysate (catalog no.20101ES60) was purchased from Yeasen Biotechnology (Shanghai) Co., Ltd. Horseradish peroxidase (HRP)-conjugated goat anti-mouse immunoglobulin G (IgG) (catalog no. SE131) and HRP-conjugated goat anti-rabbit IgG (catalog no. SE134) were procured from Solarbio Technology Co., Ltd. (Beijing). The anti-CCR10 antibody (catalog no. LS-C471078) was purchased from TechLab. The MCE antibody (catalog no. PK57329S) was acquired from Abmart Pharmaceutical Technology (Shanghai) Co., Ltd. The anti-β-actin antibody (catalog no. GB11001) was purchased from Wuhan Savier Biotechnology Co., Ltd. Lipofectamine 2000 transfection reagent (catalog no. 11668030) was purchased from Thermo Fisher Scientific. The ZO-1 antibody (catalog no. YF374758) was purchased from Invitrogen. Enzyme-linked immunosorbent assay (ELISA) kits (IL-4 [catalog no. HY4571-B], granzyme B [catalog no. HY811186-B], IFN-γ [catalog no. HY4564-B], PFP [catalog no. HY811179-B], and CCL28 [catalog no. HY0112-PB]) were purchased from Shanghai Huyu Biotechnology Co., Ltd.

### Isolation of T cells

T cells were isolated from the peripheral blood of healthy sows, and peripheral blood mononuclear cells (PBMCs) were isolated via lysis of erythrocytes as well as a washing with PBS containing 2% FBS according to the manufacturer’s instructions provided with the peripheral blood lymphocyte isolation kit. To isolate T cells from the obtained PBMCs, the nylon hair method was used for cell sorting. After sorting, the viability and quantity of the isolated T cells were evaluated by staining the cells with trypan blue. The purification of CD3^+^ T cells was verified via flow cytometry.

### Flow cytometry

The isolated T cells were washed with PBS and then filtered with 0.45 μm filters. The T cells were incubated with an APC-conjugated anti-CD3 antibody (1:500). Following incubation, the cells were subjected to two rounds of washing with PBS, followed by centrifugation at 1,500 rpm for 10 min at 4°C. The supernatant was subsequently carefully discarded. Then, the cells were resuspended in PBS. Gating strategies were implemented to exclude cellular debris and restrict the analysis exclusively to singlet cells, as identified through forward and side scatter measurements. The resulting data were subsequently analyzed via FlowJo software.

### Cell viability assay

The isolated T cells were seeded into a 96-well plate at a density of 5 × 10⁴ cells per well and then treated with PHA at a final concentration of 10 μg/mL. After 3 days, the T cells were evenly divided into two distinct groups, a negative control group and a PEDV stimulation group, with each group containing eight replicate wells, to ensure statistical reliability. The PEDV-stimulated group received an addition of inactivated PEDV QY/2016 at a multiplicity of infection (MOI) of 0.1 per well. Following a further 72-h incubation, the cytotoxic effect on the cells was quantitatively assessed via a CCK-8 assay, strictly adhering to the manufacturer’s instructions provided by Beyotime Biotechnology (Shanghai, China).

### ELISA

The isolated T cells were seeded into a six-well plate at a density of 5 × 10^6^ cells per well. These cells were then divided into two distinct experimental groups, each containing three replicate wells. One group served as the blank control, while the other was designated the PEDV stimulation group. For the PEDV group, T cells were stimulated with the PEDV QY/2016 virus at an MOI of 0.1 at 37°C in an incubator for 12 h to facilitate viral infection and a cellular response. The cell supernatant of each treatment group was collected, and the concentrations of the cytokines were measured via the following ELISA kits: Porcine granzyme B ELISA kit, Porcine perforin ELISA kit, Porcine IL-4 ELISA kit, and Porcine IFN-γ ELISA kit. Even well-grown PMECs were seeded into a 12-well plate. When the cells in the wells reached 90% confluence, the PMECs were stimulated with the PEDV QY/2016 virus at an MOI of 0.1. The supernatants were collected at 12, 24, 36, 48, and 60 h after PEDV infection, and the concentration of CCL28 was detected via ELISA. All the experiments were performed in accordance with the manufacturer’s instructions provided by Jiangsu Enzyme Industry Co., Ltd.

### Preparation of samples for RNA-seq

The isolated T cells were divided into two distinct experimental groups, each containing three replicate wells. One group served as the blank control, while the other was designated the PEDV stimulation group. For the PEDV group, T cells were stimulated with the PEDV QY/2016 virus at an MOI of 0.1 at 37°C in an incubator for 12 h. The above samples from the control group and the PEDV group were sent to Shanghai Meiji Biomedical Technology Ltd. for transcriptome sequencing, and the six samples from the two groups were named the PEDV group (inf_1, inf_2, and inf_3) and the control group (con_4, con_5, and con_6). If all the samples met the quality control criteria, all the samples were analyzed for follow-up data. All analyses were completed on the official website of the Megi biocloud platform (www.majorbio.com).

### Western blot analysis

PEDV induces the expression of CCR10 and adhesion molecules on the surface of T cells. The level of CCL28 secreted by PMECs upon PEDV infection was detected via Western blotting. The detailed procedures were as follows: the cells were lysed in cell lysis buffer (20 mM Tris-HCl [pH 7.5], 150 mM NaCl, 1 mM Na_2_EDTA, 1% Triton, 2.5 mM sodium pyrophosphate, 1 mM glycerophosphate, 1 mM Na_3_PO_4_, 1 g/mL leupeptin, and 1 mM phenylmethylsulfonyl fluoride), and the supernatant produced by centrifugation at 12,000 rpm for 15 min at 4°C was collected. Protein concentrations were measured with the Bio-Rad protein assay. Different cellular proteins were detected with anti-CCR10, anti-CCL28, anti-paxillin, vinculin, FAK, Talin-1, and α-actinin antibodies at a dilution of 1:1,000. The primary antibodies were detected with horseradish peroxidase-conjugated goat anti-rabbit antibodies. After extensive washing with TBST, immunoreactive bands were detected via film exposure after the ECL reaction.

### siRNA-mediated knockdown

According to the gene sequence (NM_001044563.1) of the porcine chemokine receptor CCR10 registered in the GeneBank, three corresponding CCR10 siRNAs and a negative control (NC) with no homology to the target genes were synthesized by Sangon Bioengineering (Shanghai, China), as shown in Table 1. T cells were seeded in 12-well culture plates at 5 × 10^5^ cells/well before transfection. The above siRNAs were then transfected into T cells via the Lipofectamine 2000 transfection reagent according to the manufacturer’s instructions. Six hours after transfection, the transfected cells were infected with the PEDV QY2016 strain at an MOI of 0.1 for 24 h at 37°C. Moreover, a blank control group and a PEDV infection group were designed. The efficiency of these siRNAs and the expression of adhesion molecules on T cells were characterized by Western blot and RT-qPCR.

**TABLE 1:** Sequences of the siRNAs used in this study

Gene name	Sense sequence	Anti-sense sequence
CCR10-45	GGG ACU AUG AAG AGG CAU A	UAU GCC UCU UCA UAG UCC C
CCR10-802	GUU GGA UAC UGC UGA CCU A	UAG GUC AGC AGU AUC CAA C
CCR10-909	GCC UCA ACC CAG UGC UCU A	UAG AGC ACU GGG UUG AGG C
NC	UUC UCC GAA CGU GUC ACG U	ACG UGA CAC GUU CGG AGA A

### RT-qPCR

PEDV induces the expression of CCR10 on the surface of T cells. The level of CCL28 secreted by PMECs upon PEDV infection was detected via RT-qPCR. According to the sequences of porcine CCL28 (NM_001024695.1), CCR10 (NM_001044563.1), and β-actin (XM_003357928.4) recorded in GenBank, the corresponding fragment detection primers for the gene were designed via Primer Premier 5 software and sent to Sangon Bioengineering (Shanghai) Co., Ltd. for synthesis. The specific primers used are shown in Table 2. The detailed RT-qPCR procedures were as follows: total RNA was obtained from each group and then reverse-transcribed into cDNA using oligo (dT) as the primer. The RT-qPCR program was as follows: 95°C for 120 s, followed by 35 cycles (5 s at 95°C and 5 s at 49.5°C), and finally cooling at 72°C for 25 s. The fold change was determined via the 2^−ΔΔ*t*^ method with the housekeeping gene β-actin for normalization.

**TABLE 2:** PCR primers used to amplify partial genes encoding CCL28, CCR10, and β-actin

Gene name	Primer sequence (5′→3′)	Product size (bp)
CCL28	Forward: TGCTGCACTGAGGTTTC Reverse: GATGATTATGAGGGCTGAC	151
CCR10	Forward: GGATCTGGCACTGCTGGTGA Reverse: GGAGCCGAACAGGAAGAAAGG	192
β-actin	Forward: CTGGCATTGTCATGGACTCT Reverse: GCGATGATCTTGATCTTCAT	276

### Transmigration assay

The PMECs used in this study were cultured in DMEM containing 5 mg/L bovine insulin, 5 mg/L hydrocortisone, 10 ng/mL epidermal growth factor (EGF), 50 mg/L gentamicin, 10% FBS, and 2% double antibiotics were placed in a 37°C incubator with 5% CO_2_. The lower sides of 5-μm-pore-size polycarbonate filters of a 12-well transwell plate (Corning Incorporated Costar, Cambridge, MA, USA) were seeded with PMECs. The specific inoculation method was as follows: well-grown ninth-generation PMECs were routinely digested and counted. They were inoculated into the upper chamber of a Transwell system at a concentration of 5 × 10^5^ cells/mL, with 200 μL of complete medium added to the upper chamber and 800 μL of complete medium added to the lower chamber. The transepithelial electrical resistance (TEER) of the PMECs was measured via a cell resistance meter on days 0, 2, 4, 6, 8, and 10. After culturing for 6–8 days, the polycarbonate membrane in the chamber was removed via a surgical blade and ophthalmic tweezers, and then mounted onto a slide. The confluence of the PMEC monolayers was assessed by staining the tight junction protein ZO-1 with an anti-ZO-1 antibody. The isolated T cells were seeded into the upper chamber of PMECs. The recombinant chemokine CCL28 (500 ng/mL) was added to the lower chambers of several filters. After 12 h of migration at 37°C with 5% CO_2_, T cells were collected via centrifugation of the lower-chamber medium, quantified via trypan blue staining, and statistically analyzed.

### Statistical analysis

All the data were plotted via GraphPad Prism 8.0 software, and the statistical significance of differences between groups was examined via one-way analysis of variance (ANOVA). The results are expressed as the means ± standard errors. When *P* < 0.05, the difference is significant (*); when *P* < 0.01, the difference is extremely significant (**); and when *P* < 0.001, the difference is very significant (***).

## RESULTS

### PEDV inhibits the T-cell immune response

**Fig 1:**
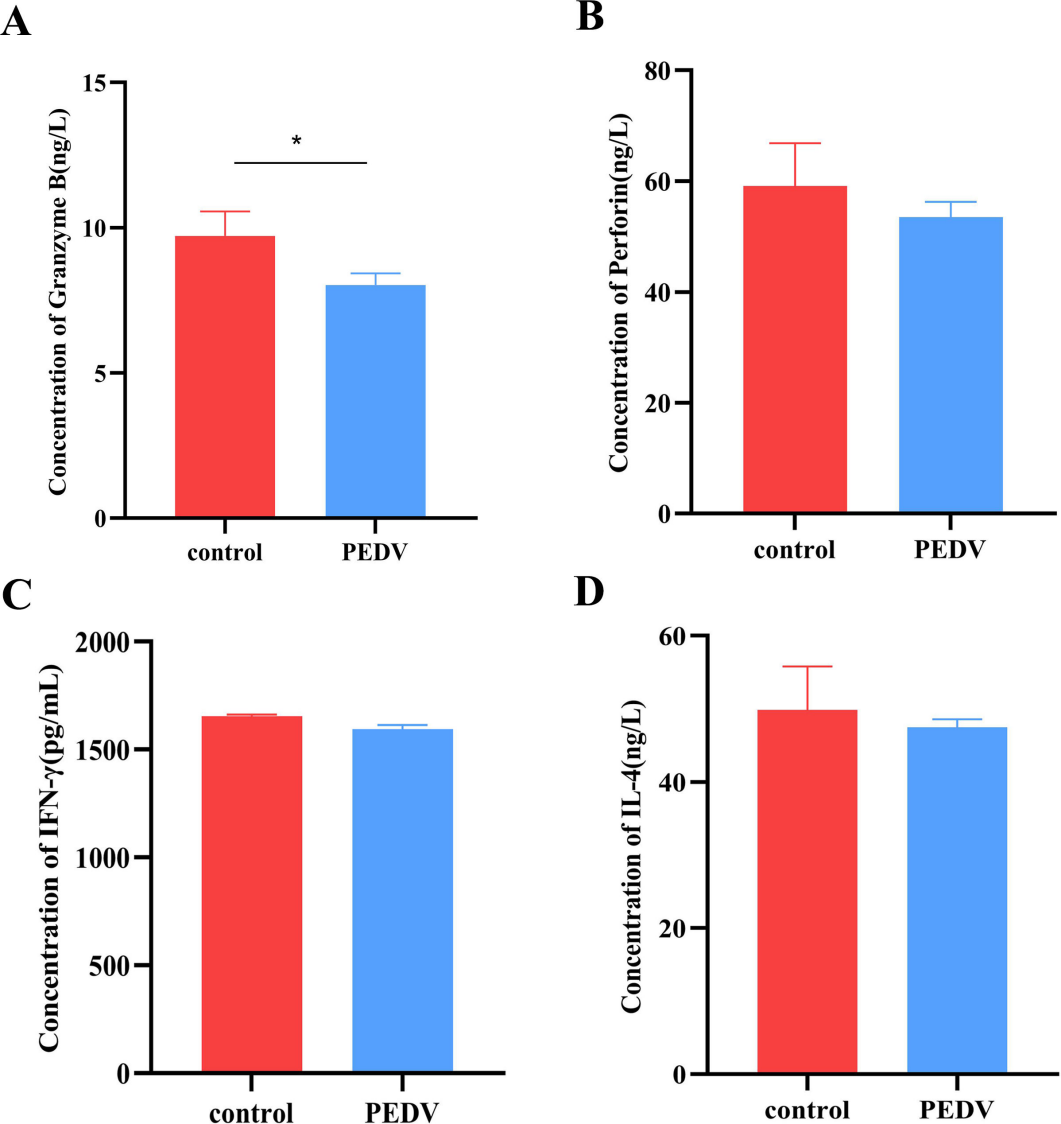
Levels of granzyme B, perforin, IFN-γ, and IL-4 in T cells in response to PEDV infection. T cells were collected after PEDV virulent (QY2016) infection at 12 h, and the secretion levels of granzyme B (**A**), perforin (**B**), IFN-γ (**C**), and IL-4 (**D**) were detected via ELISA. * indicates a significant difference between the infection group and the control group (*P* < 0.05). Each group consisted of three repetitions.

The flow cytometry results revealed that the purity of the isolated T cells reached 95.1% (Fig. S1A), which was suitable for subsequent experiments. T cells activated by PHA stimulation were divided into a negative control group and a PEDV stimulation group (MOI = 0.1). The OD_450_ value was detected via the CCK-8 method. The results revealed that the OD_450_ value of T cells stimulated with PEDV for 72 h was significantly greater than that of the negative control group, indicating that the isolated T cells had good activity (Fig. S1B). To detect the effects of PEDV infection on T-cell function further, ELISA kits were used to detect the secretion levels of IL-4, IFN-γ, granzyme B, and perforin. After T cells were stimulated with PEDV for 12 h, the content of granzyme Bsecreted by T cells decreased significantly (Fig. 1A), and the secretion of perforin, IFN-γ, and IL-4 also tended to decrease (Fig. 1B through D), but the difference was not significant. These results suggest that PEDV stimulation of T cells may inhibit the immune regulatory function of T cells.

### Analysis of global transcript expression in porcine T cells via RNA-seq

**Fig 2:**
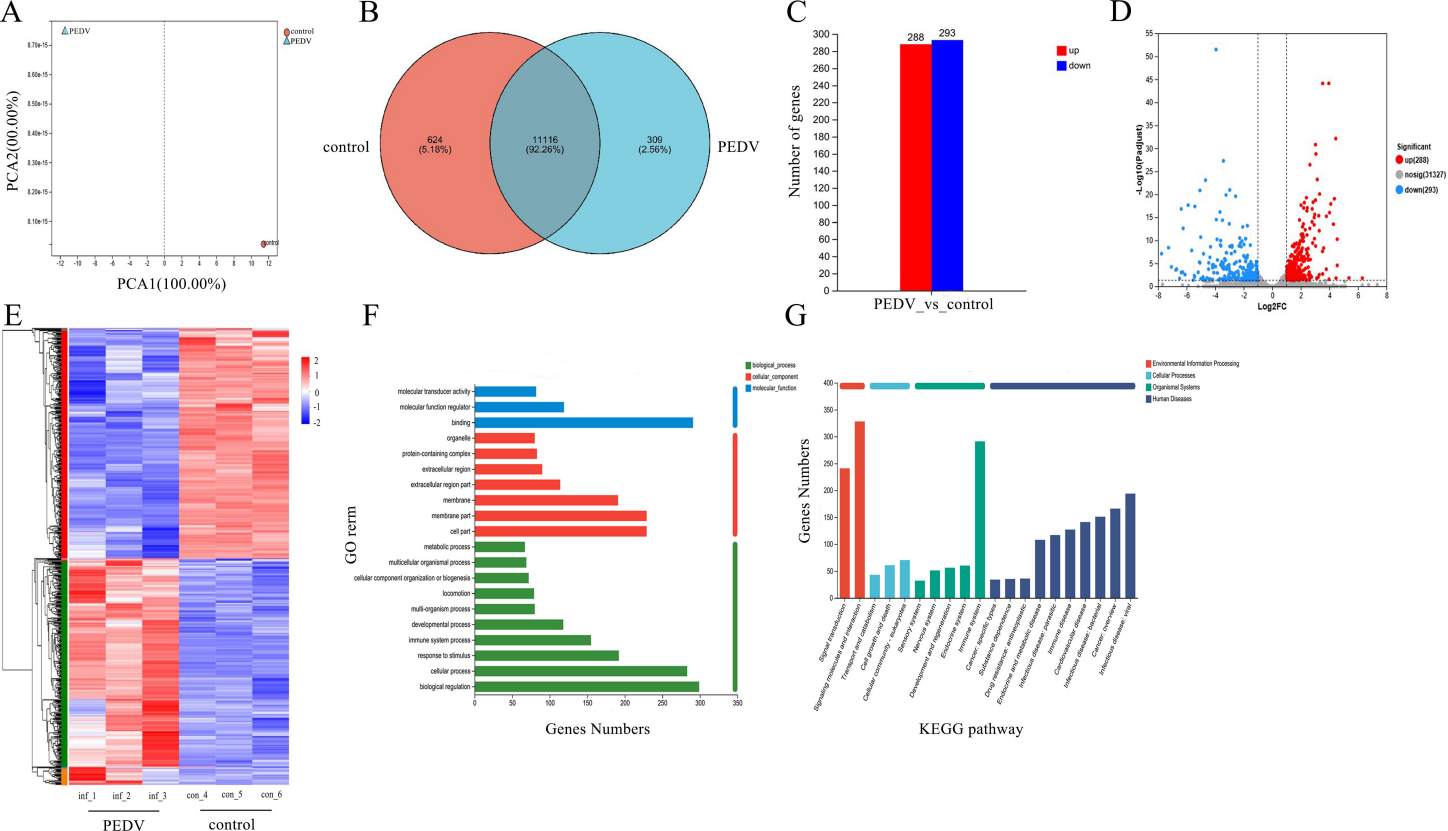
T-cell transcriptomic data analysis. (**A**) PCA revealed disparities between different groups. (**B**) Venn diagram. Red circles indicate control group-specific genes, blue circles indicate group-specific genes, and gray areas indicate genes shared between the two groups. The bar chart (**C**) and volcano plot (**D**) show significant differences, among which 288 genes significantly increased and 293 genes significantly decreased. (**E**) Heatmap of DEGs associated with T cells. (**F**) GO enrichment analysis of differentially expressed genes in T cells. The *x*-axis represents the number of differentially expressed genes, and the *y*-axis represents the GO terms. (**G**) Top 20 signaling pathways. A smaller *P* value suggests that the enrichment was more significant. The *y*-axis represents the functional descriptions of the KEGG terms.

A total of six libraries of T cells from the untreated group and the PEDV-stimulated group were sequenced. After the removal of invalid data from 41.4 to 57.6 million raw reads, more than 98.73% of the data were considered valid and were collected for each library. The Q20% (sequencing error rate <0.01) was greater than 97.61%, and the Q30% (sequencing error rate <0.001) was greater than 93.26% (Table S1). Principal component analysis (PCA) revealed disparities between different groups as well as correlations within each group, as illustrated in Fig. 2A. These comprehensive data collectively validated the reproducibility of our samples. There were a total of 581 genes whose expression significantly differed, among which the expression of 288 genes increased significantly and that of 293 genes decreased significantly in PEDV-infected T cells. The differentially expressed genes (DEGs) were associated with multiple biological processes, and the leukocyte transendothelial migration and chemokine signaling pathways presented the greatest enrichment according to the Gene Ontology (GO) and Kyoto Encyclopedia of Genes and Genomes (KEGG) enrichment analyses.

The Venn analysis results revealed 11,116 common genes, 624 genes unique to the control group, and 309 genes unique to the infection group (Fig. 2B). The differences in the expression of the 11,116 common genes were analyzed. The analysis conditions were a fold change ≥2 and *P* < 0.05. The bar chart and volcano plot revealed 581 genes whose expression significantly differed, among which 288 genes presented significant increases, and 293 genes presented significant decreases (Fig. 2C and D). The 581 genes whose expression significantly differed were subsequently grouped into a gene set and subjected to cluster analysis. A heatmap revealed the specific upregulated and downregulated genes among the 581 significantly different genes between the PEDV stimulation group and the control group (Fig. 2E). The functional annotation analysis of 581 significantly differentially expressed genes was conducted via the GO database and the KEGG database. GO annotation analysis revealed the biological function of “locomotion” (Fig. 2F). In the KEGG annotation analysis, functional annotations such as “signal transduction” and “signaling molecules and interaction” were discovered (Fig. 2G), which might be related to cell migration.

### PEDV induces the expression of CCR10 on the surface of T cells

**Fig 3:**
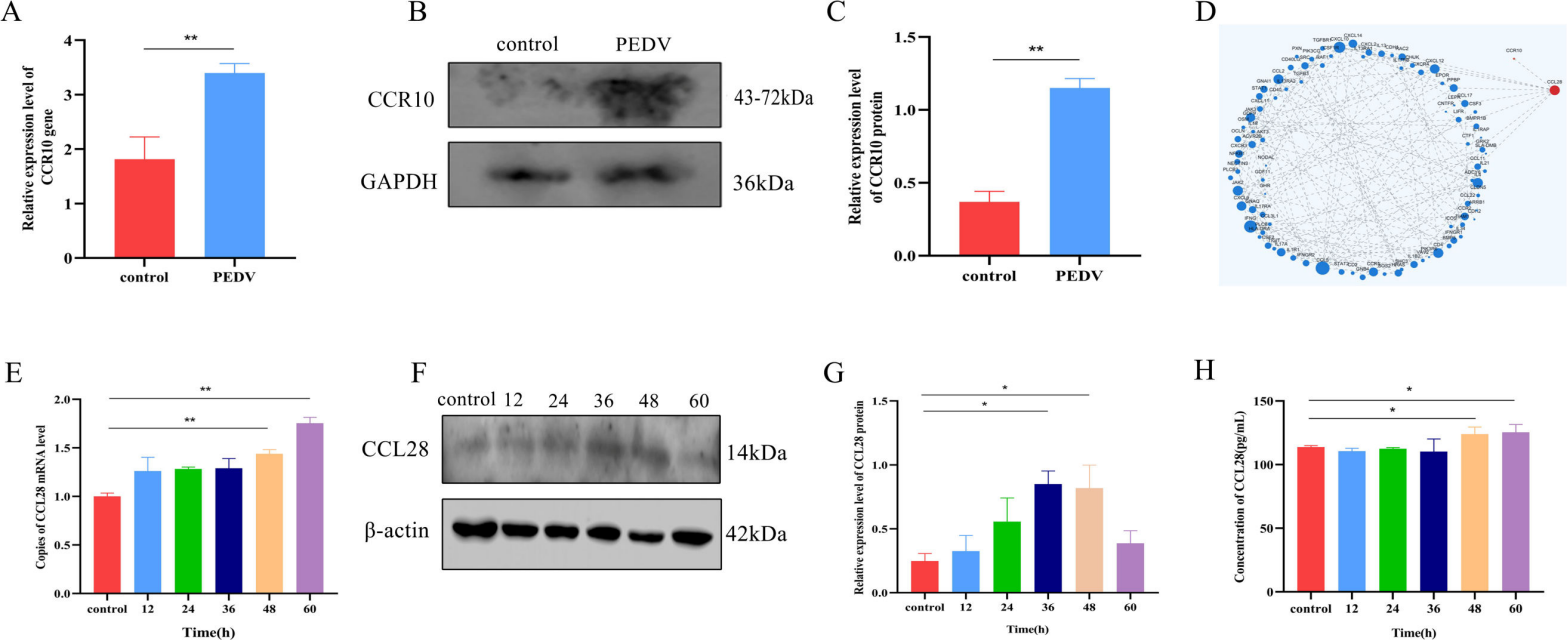
Expression of CCR10 on T cells and CCL28 in PMECs after PEDV infection. (**A**) Expression levels of CCR10 in T cells from transcriptome data. (**B**) The expression levels of CCR10 in T cells from the PEDV-QY2016-infected and negative control groups were quantitatively determined via Western blotting. (**C**) The relative protein expression levels of CCR10 after normalization to internal parameters. (**D**) Protein-protein interaction (PPI) network analysis. (**E and F**) The levels of the CCL28 gene and protein in breast epithelial cells infected with PEDV at different time points were detected by qPCR (**E**) and Western blotting (**F**), respectively. (**G**) The relative expression levels of the CCL28 protein after internal parameter normalization. * represents a significant difference relative to the control group (*P* < 0.05), ** represents an extremely significant difference relative to the control group (*P* < 0.01). (**H**) The level of CCL28 in the supernatant of PMECs stimulated with the PEDV QY/2016 virus was detected via ELISA.

RNA-seq analysis revealed that PEDV significantly increased the expression of CCR10 on the surface of T cells (Table S2). The results of the RNA-seq data were verified via RT-qPCR and Western blotting. As shown in Fig. 3A through C, the expression pattern of CCR10 was consistent. These results confirm the reliability of our RNA-seq data. The results of the personalized protein-protein interaction network analysis subsequently revealed a protein interaction between CCR10 and CCL28 (Fig. 3D). The expression of CCL28 in PMECs at different time points after PEDV infection was subsequently detected via RT-qPCR and Western blotting. The RT-qPCR results revealed that the CCL28 gene in PMECs stimulated with PEDV significantly increased after 48 h (Fig. 3E). The Western blot results indicated that the expression of CCL28 in PMECs significantly increased after 36 h of PEDV infection and peaked at 48 h (Fig. 3F and G). ELISA results revealed that the concentration of CCL28 in the supernatant significantly increased after PEDV was used to stimulate PMECs for 48 h (Fig. 3H).

### Common DEGs in T cells regulated by PEDV are associated with adhesion molecules

**Fig 4:**
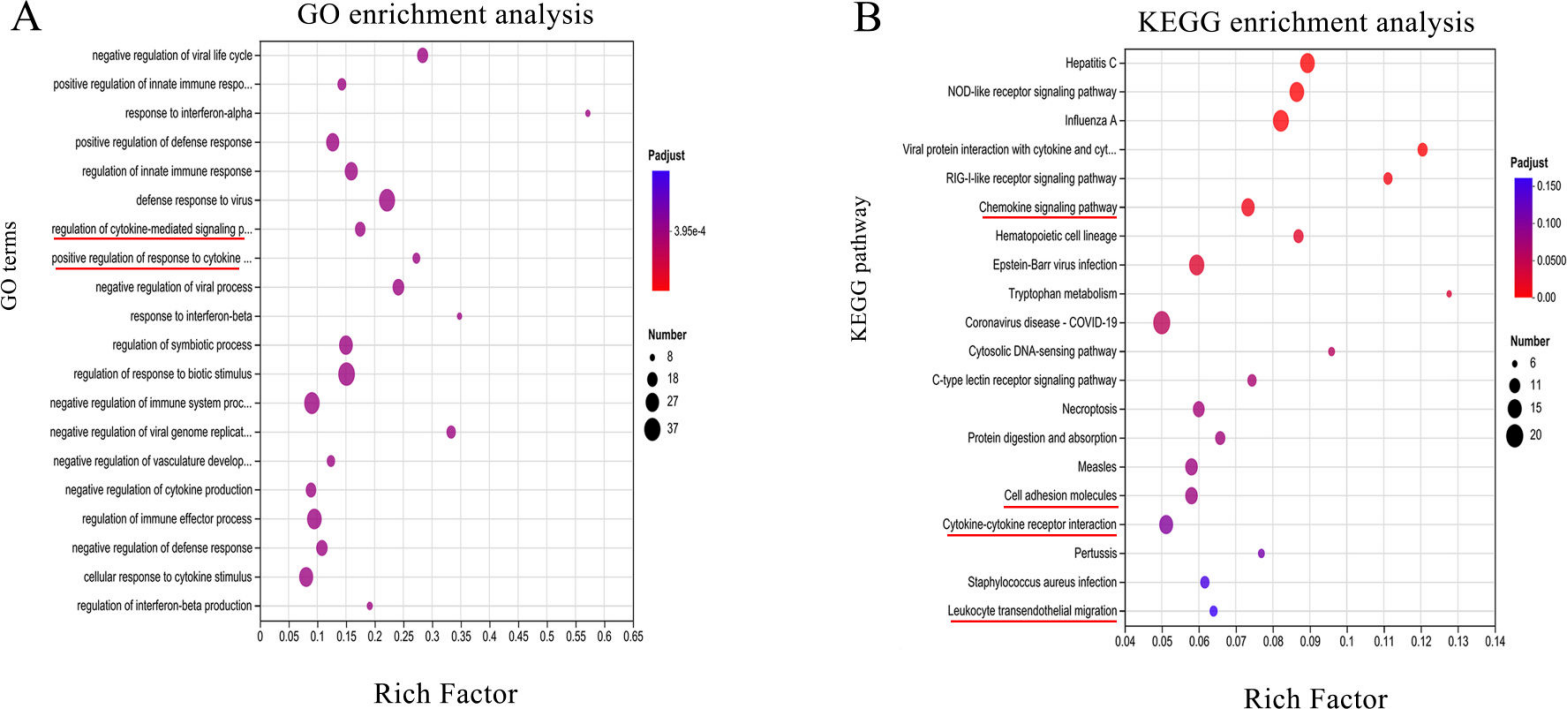
Gene enrichment analysis. (**A**) GO enrichment analysis of differentially expressed genes in T cells. (**B**) Top 20 signaling pathways. The size and color of the circles represent the number of genes and the range of *P*-adjusted values, respectively.

**Fig 5:**
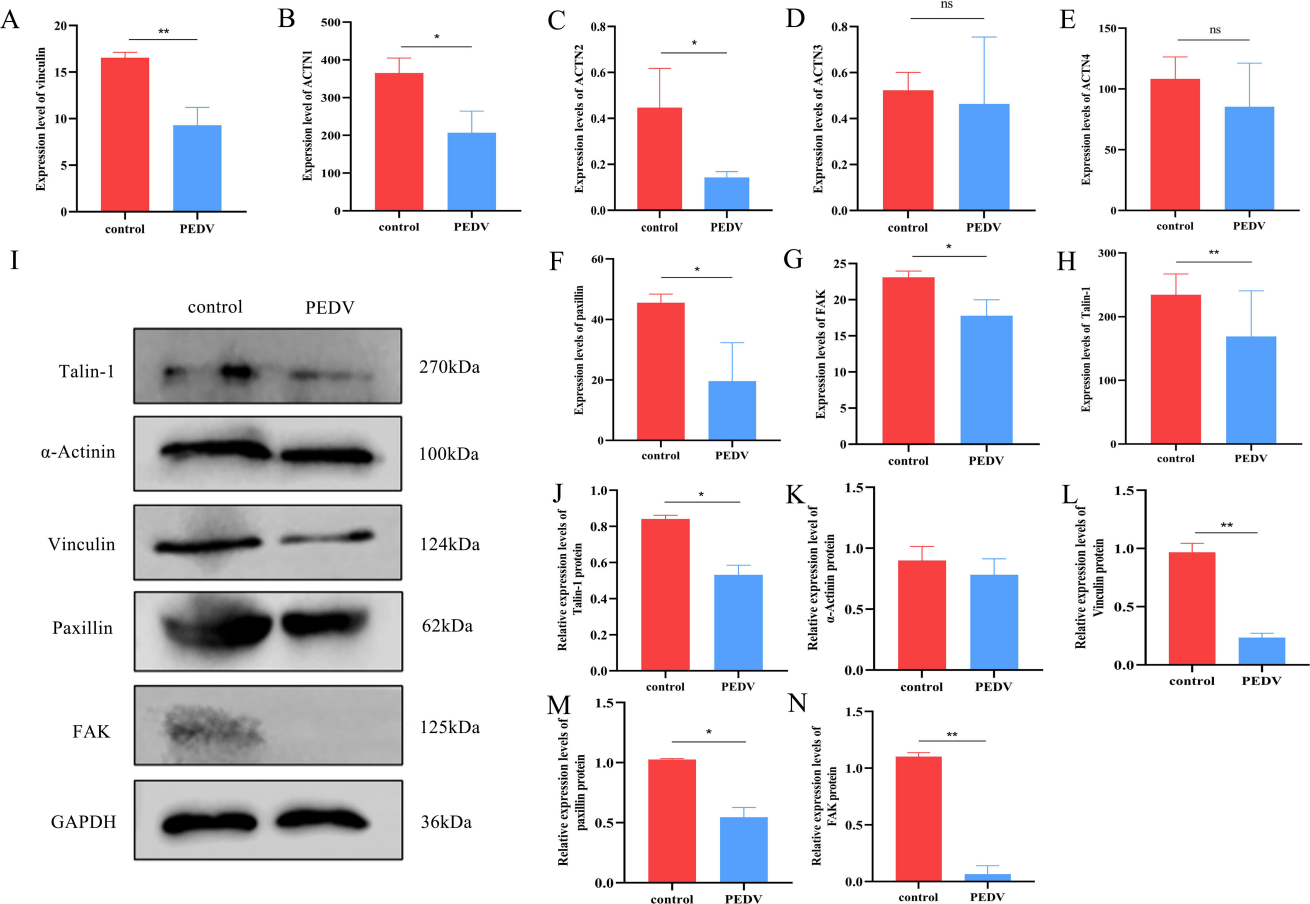
Expression of adhesion proteins in T cells in response to PEDV infection. (**A–G**) Expression levels of vinculin, ACTN1, ACTN2, ACTN3, ACTN4, paxillin, and FAK in T cells from transcriptome data. (**H–N**) The band intensity shown in panel **I** was analyzed with ImageJ software.

To better understand the functions of DEGs induced by PEDV, a total of 581 genes whose expression significantly differed were subjected to GO clustering analysis and KEGG clustering analysis. The GO enrichment analysis results indicated that among the top 20 enriched pathways, the regulation of cytokine-mediated signaling and positive regulation of the response to cytokines were prominently clustered (Fig. 4A). In the KEGG enrichment analysis, the top 20 enriched pathways included leukocyte transendothelial migration, the chemokine signaling pathway, cell adhesion molecules, and cytokine-cytokine receptor interactions (Fig. 4B). The genes clustered into four pathways associated with adhesion molecules. For example, vinculin, α-actinin, paxillin, FAK (PTK2), and Talin-1. The RNA-seq data revealed that upon stimulation with PEDV, the expression levels of the vinculin, paxillin, FAK, and talin-1 genes in T cells markedly decreased (Fig. 5A through H; Table S3). To validate the RNA-seq data, Western blotting was performed on some DEGs associated with adhesion molecules. The results indicated that upon stimulation with PEDV, T cells presented notable decreases in the protein levels of vinculin, paxillin, FAK, and talin-1. Additionally, the level of the α-actinin protein also tended to decrease; however, this change did not reach statistical significance (Fig. 5I and J).

### CCR10 enhances the transcellular migration of T cells across PMECs

**Fig 6:**
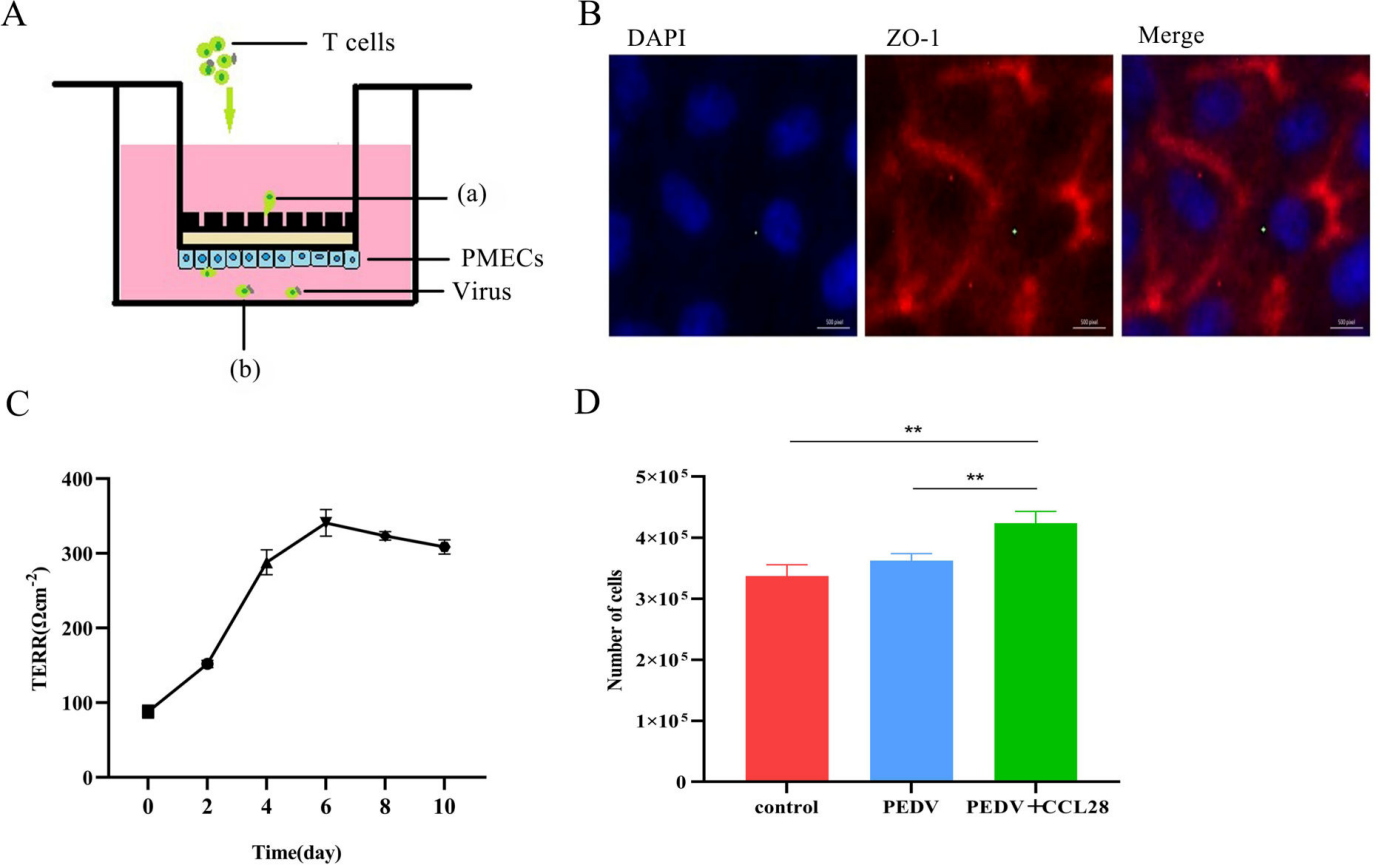
CCL28 induces PEDV-carrying T cells to migrate across PMECs. (**A**) Schematic representation of the transwell migration assay. T cells infected with PEDV or the controls were added to the upper chamber in the presence or absence of the chemokine CCL28 in the lower chamber. (**B**) IFA was used to detect the expression of ZO-1 in a coculture model of T cells and PMECs. Blue, DAPI; red, ZO-1. (**C**) Changes in Transwell chamber resistance after PMEC seeding. (**D**) Counting the number of cells retrieved from the lower chamber enabled the determination of the number of T cells that fully migrated through the PMECs.

To investigate the role of T cells in the migration process, this study established a coculture model of T cells and PMECs (Fig. 6A). The IFA results demonstrated that the ZO-1 antibody specifically bound to the target protein. Red fluorescence signals were observed between the cells, suggesting the successful formation of well-defined tight junctions among the PMECs (Fig. 6B). After the PMECs were seeded into the Transwell chambers, their transepithelial electrical resistance (TEER) was measured via a cell resistance meter on days 0, 2, 4, 6, 8, and 10. The results showed that by day 4 of culture, the resistance value had reached the minimum requirement (300 Ω·cm²), reaching its peak on day 6, and between days 6 and 10, the resistance of the mammary epithelial cells met the experimental requirements (>300 Ω·cm²) (Fig. 6C). The number of cells that migrated to the lower chamber while maintaining the integrity of the single-layer PMECs was subsequently quantified. This was achieved by adding CCL28 protein (500 ng/mL) to the lower chamber. These findings indicated that the addition of the CCL28 protein effectively induced a greater number of virus-carrying T cells to traverse the PMECs and migrate into the lower chamber of the well (Fig. 6D).

### CCR10 regulates paxillin/FAK-mediated T-cell migration

**Fig 7:**
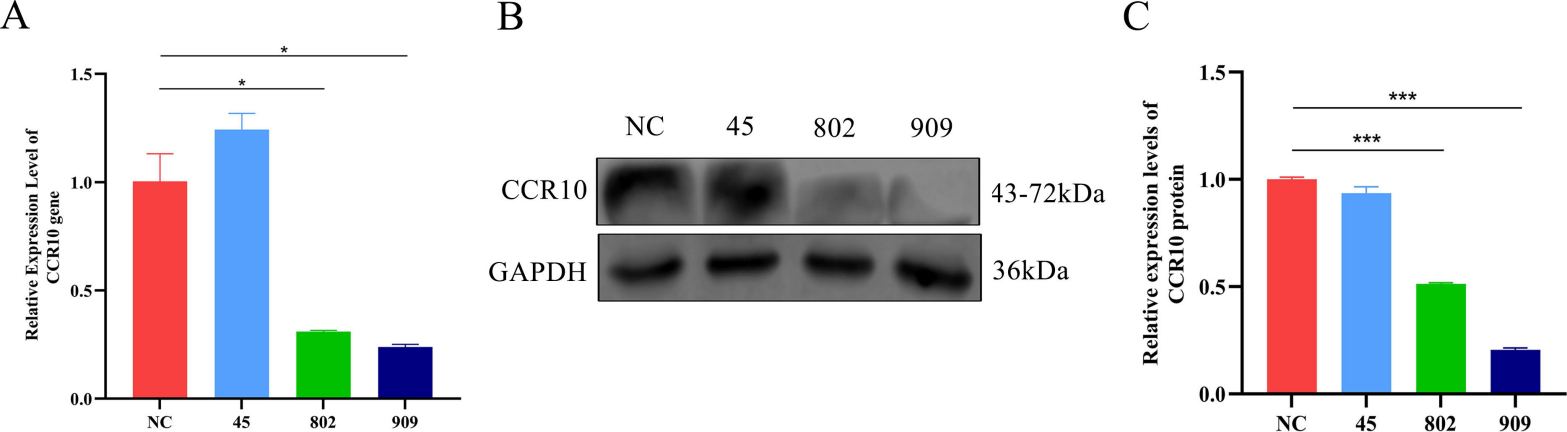
Screening siRNAs targeting CCR10. T cells in 12-well plates were transfected with the indicated siRNA using the transfection reagent Lipofectamine 2000 according to the specifications. Twelve hours after treatment, the nucleotides and the supernatants from the T cells were subjected to RT-qPCR (**A**) and Western blotting (**B**) to analyze the effects of the siRNAs. (**C**) The band intensity shown in panel **B** was analyzed with ImageJ software.

**Fig 8:**
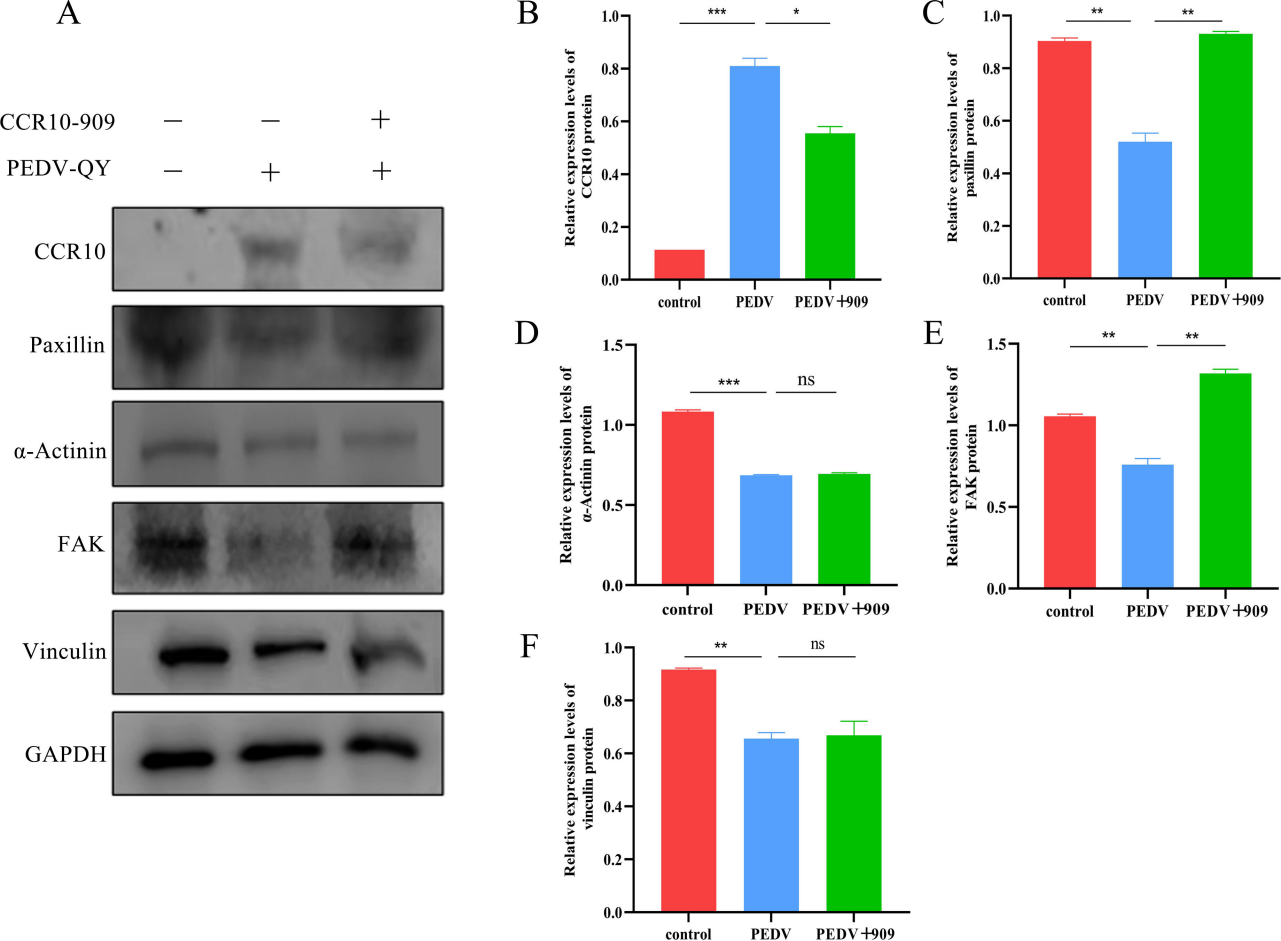
Effect of CCR10 inhibition on related adhesion proteins in T cells in response to PEDV infection. (**A**) T cells in 12-well plates were transfected with the indicated siRNA using the transfection reagent Lipofectamine 2000 according to the manufacturer’s specifications. Four hours after treatment, the transfected cells were infected with the PEDV-QY2016 strain at an MOI of 0.1 for 24 h at 37°C. Then, the T cells were collected and subjected to Western blotting. (**B–F**) The band intensity shown in panel **A** was analyzed with ImageJ software.

T cells were transiently transfected with siRNAs (CCR10_45, CCR10_802, and CCR10_909) targeting CCR10 as well as the control siRNA. Among these siRNAs, the CCR10_909 siRNA apparently reduced the expression of CCR10, as determined by RT-qPCR and Western blotting (Fig. 7A through C). Therefore, we used siRNA CCR10_909-mediated knockdown to address the specific role of CCR10 in T-cell migration. Subsequently, we aimed to determine whether the inhibition of CCR10 by CCR10_909 impacts proteins associated with cell adhesion, such as paxillin, FAK, vinculin, and α-actinin. T cells were classified into three groups: a negative control group, an NC interference-PEDV stimulation group, and a CCR10_909 interference-PEDV stimulation group. Western blotting was used to detect proteins (Fig. 8A). These findings indicated that upon PEDV infection, the expression of CCR10 increased significantly (Fig. 8B). Conversely, the expression levels of paxillin, FAK, vinculin, and α-actinin all tended to decrease. Transfection with CCR10_909 led to notable increases in the protein levels of paxillin and FAK. However, no significant changes in the protein levels of vinculin or α-actinin were detected following transfection (Fig. 8C through F).

## DISCUSSION

PED is an acute and contagious gastrointestinal disorder in pigs that is induced by PEDV. PEDV predominantly affects neonatal piglets and ranks among the most critical pathogens affecting the swine industry. It has caused substantial economic losses in the swine farming sector both in China and globally (1–3, 7). At present, most of the research regarding PED has focused on pathogen isolation and identification, epidemiological investigations, drug development, and vaccine research (17–21). In contrast, relatively few studies have investigated the mechanism of PEDV transmission within the host. PEDV has multiple transmission pathways. In addition to being transmitted via the digestive and respiratory tracts, recent research has indicated that sow colostrum also serves as a crucial route for PEDV transmission (5, 10, 11). Our recent study revealed that virus-carrying T cells are present in the colostrum of sows and can cause PEDV infection in piglets (11). However, the origin of virus-carrying T cells in the colostrum remains unclear.

This study revealed that the levels of IL-4, IFN-γ, granzyme B, and perforin secreted by T cells tended to decrease after PEDV infection, indicating that PEDV infection might inhibit the immune function of T cells. Moreover, our previous research revealed that PEDV can be detected in T cells stimulated with PEDV after 12 h. PEDV establishes latent infection in T cells, inhibits interferon secretion, and evades immune clearance. This strategy may prolong the virus’s survival time within the host, providing a time window for regulating T-cell migration, ultimately facilitating the vertical transmission of the virus to piglets via colostrum.

Here, we studied the transcriptome of T cells to understand gene expression profiles under PEDV infection. A total of 581 DEGs were involved in multiple biological processes. To explore the functions of DEGs that were differentially expressed in response to PEDV, GO and KEGG clustering analyses were conducted on 581 significantly differentially expressed genes. GO enrichment analysis revealed that among the top 20 enriched pathways, regulation of cytokine-mediated signaling and positive regulation of the response to cytokines were prominently clustered. KEGG enrichment analysis revealed that the top 20 enriched pathways included leukocyte transendothelial migration, the chemokine signaling pathway, cell adhesion molecules, and cytokine-cytokine receptor interactions, with genes in four pathways associated with adhesion molecules. RNA-seq data revealed a marked decrease in the expression levels of the vinculin, paxillin, FAK, and talin-1 genes in T cells upon PEDV infection, which was validated by Western blotting, which revealed notable reductions in the corresponding protein levels. To our knowledge, this study is the first to reveal the suppression of adhesion molecule-related genes by PEDV infection. In the following, we focused on the relationship between adhesion molecules and T-cell migration during PEDV infection.

Numerous studies have demonstrated that mammary epithelial cells can generate substantial quantities of chemokines, which effectively facilitate the migration of immune cells across the mammary epithelium into the colostrum. Specifically, among these chemokines, CCL2, which is secreted by mammary epithelial cells, plays a pivotal role in guiding monocytes to home to mammary epithelial cells. Moreover, CXCL1, another chemokine secreted by mammary epithelial cells, can effectively promote the migration of neutrophils across the mammary epithelium and into the colostrum (22). The chemokine CCL28 was first identified by Wang et al. (23). It belongs to a distinct chemokine family characterized by unique features in terms of gene location and protein sequence (23, 24). CCL28 is expressed by various mucosal epithelial cells. In addition to its function in coordinating lymphocyte trafficking, CCL28 is also critical for modulating the homing capacity of lymphocytes. It has been reported that the secretion of CCL28 by PMECs significantly affects the quantity of IgA^+^ B cells during pregnancy (25). We previously reported that our previous reports indirectly demonstrated that the interaction between CCR10 and CCL28 can facilitate the migration of T cells across the mammary epithelium (11). In this study, the role of CCR10/CCL28 in the migration of T cells across the mammary epithelium during PEDV infection was first confirmed through a PMEC and T-cell coculture model.

T-cell migration is achieved by regulating the release of intracellular chemokines at interaction sites between the mammary epithelium and T cells through PEDV, creating a microenvironment conducive to migration. Our research addressed the important topic of how viral stimulation of intrinsic T-cell signals and the interaction between them affect the migration of virus-harboring T cells across the mammary epithelium. On the basis of the transcriptome data, we postulate that the interaction between CCR10, which is expressed on the surface of T cells activated by PEDV, and CCL28 secreted by PMECs facilitates the transepithelial migration of T cells. This process is intricately associated with the adhesion molecules (FAK and paxillin) expressed by T cells. The dynamic interplay between FAK and paxillin facilitates the recycling and translocation of focal adhesion proteins, consequently affecting cell motility and adhesion (26, 27). Luscinskas et al. presented evidence that the silencing of both paxillin and FAK impeded neutrophil migration without affecting neutrophil adhesion (28). In the present study, PEDV stimulation of T cells significantly downregulated the expression levels of paxillin and FAK while concurrently increasing the number of migrating T cells. This finding implies that PEDV may enhance T-cell migration by modulating the expression of paxillin and FAK, thereby influencing T-cell adhesion. Through further application of the coculture model of T cells and PMECs and siRNA technology, it was found that upon inhibiting the expression of CCR10 on the surface of T cells, the protein expression of both paxillin and FAK was upregulated. This could be attributed to the fact that CCR10 interacts with CCL28 to activate FAK and paxillin, thus facilitating the transmembrane migration of T lymphocytes.

### Conclusion

PEDV stimulation dysregulates T-cell immunity, suppressing the expression of the cytotoxic molecule granzyme. The deep transcriptomic profiles of PEDV-stimulated infected T cells were systematically studied. RNA-seq revealed that PEDV-infected T cells presented significantly upregulated CCR10 expression and downregulated the expression of cytoskeletal regulators (vinculin, α-actinin, paxillin, FAK, and talin-1). Mechanistically, PEDV induces CCL28 secretion from PMECs, which drives the migration of T cells carrying PEDV via the CCR10/CCL28 axis activation. Silencing CCR10 attenuated T-cell-induced PEDV migration via paxillin/FAK pathway modulation, demonstrating the exploitation of host transcriptional reprogramming by PEDV. These findings reveal a novel mechanism of PEDV transmission from sows to neonatal piglets and identify CCR10/CCL28 and cytoskeletal signaling as potential targets for blocking colostrum-borne infection.

## Data Availability

The data sets used and analyzed during the current study are archived at Hebei Agricultural University.

## SUPPLEMENTAL MATERIAL

Supplemental material jvi.00024-26-s0001.docx Fig. S1; Tables S1 to S3.

ASM does not own the copyrights to Supplemental Material that may be linked to, or accessed through, an article. The authors have granted ASM a non-exclusive, world-wide license to publish the Supplemental Material files. Please contact the corresponding author directly for reuse.
