# Supplementary material for: PEDV regulates trans-mammary epithelial migration of T cells in a CCR10/CCL28-dependent manner
Source: J Virol. 2026 Mar 20;100(4):e00024-26. doi: 10.1128/jvi.00024-26 (PMC13098241; doi:10.1128/jvi.00024-26)
Supplement: Supplemental material — Fig. S1; Tables S1 to S3. [file jvi.00024-26-s0001.docx]

**Supplementary Data**

**SFig. 1. Purity and activity of T cells.**

**
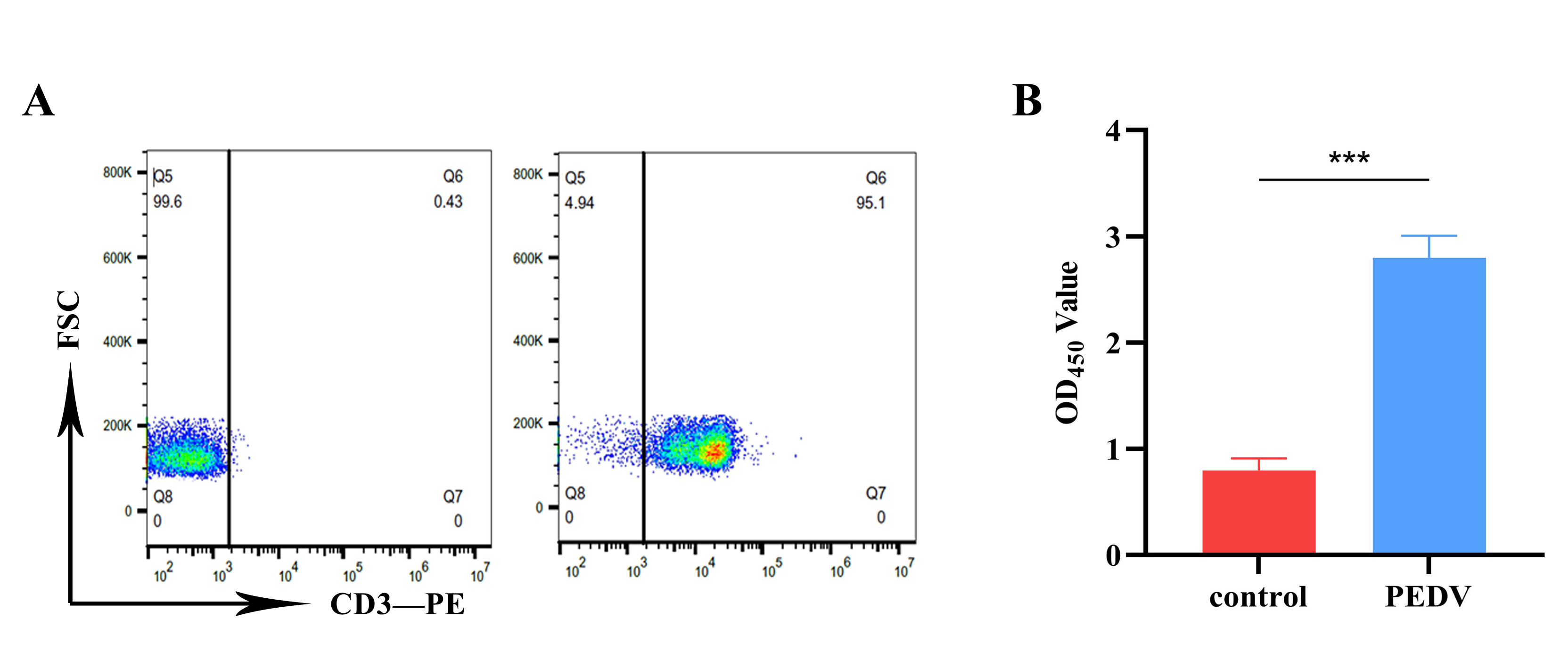
**

1. Flow cytometry was used to determine the purity of the T cells. **B**. A CCK8 assay was used to assess the viability of T cells.

**Supplementary Table 1. Original RNA sequencing data.**

Supplementary Table1. Allegation data sheet.

| sample | Raw reads | Raw Bases | Clean reads | Clean bases | Error Rate（%） | Q20（%） | Q30（%） | GC  content（%） |
| --- | --- | --- | --- | --- | --- | --- | --- | --- |
| inf_1 | 49553502 | 7482578802 | 49079426 | 7296167237 | 0.0253 | 97.92 | 93.96 | 51.68 |
| inf_2 | 44037192 | 6649615992 | 43637616 | 6481995482 | 0.0253 | 97.91 | 93.89 | 50.96 |
| inf_3 | 57666202 | 8707596502 | 56934204 | 8432047720 | 0.0255 | 97.83 | 93.8 | 50.11 |
| Con_4 | 41447084 | 6258509684 | 40940396 | 6069898566 | 0.026 | 97.61 | 93.26 | 50.55 |
| Con_5 | 47806776 | 7218823176 | 47300906 | 7015601702 | 0.0254 | 97.87 | 93.84 | 50.64 |
| Con_6 | 46732090 | 7056545590 | 46288270 | 6911933733 | 0.0252 | 97.93 | 94.07 | 53.54 |

**Supplementary Table 2. Expression levels of CCR10 in the transcriptome data.**

Supplementary Table 2. Expression levels of CCR10 in the transcriptome data.

|  | inf_1 | inf_2 | inf_3 | con_4 | con_5 | con_6 |
| --- | --- | --- | --- | --- | --- | --- |
| CCR10 | 3.40 | 3.57 | 3.22 | 1.50 | 1.66 | 2.28 |

**Supplementary Table 3. Expression of related adhesion genes in the transcriptome data.**

Supplementary Table 3 Expression of related adhesion genes in the transcriptome data.

|  | Gene Description | inf_1 | inf_2 | inf_3 | con_4 | con_5 | con_6 | Infect  (avg) | Control  (avg) |
| --- | --- | --- | --- | --- | --- | --- | --- | --- | --- |
| VCL | vinculin | 9.23 | 7.40 | 11.24 | 16.97 | 16.78 | 15.88 | 9.29 | 16.54 |
| ACTN4 | actinin alpha 4 | 125.32 | 74.96 | 55.86 | 100.55 | 95.30 | 128.98 | 85.38 | 108.28 |
| ACTN2 | actinin alpha 2 | 0.12 | 0.14 | 0.17 | 0.25 | 0.53 | 0.56 | 0.14 | 0.45 |
| ACTN1 | actinin alpha 1 | 225.76 | 252.40 | 142.26 | 341.13 | 343.16 | 411.27 | 206.81 | 365.19 |
| ACTN3 | actinin alpha 3 | 0.30 | 0.80 | 0.29 | 0.46 | 0.50 | 0.61 | 0.46 | 0.52 |
| PXN | paxillin | 33.58 | 16.49 | 8.70 | 42.24 | 47.06 | 47.33 | 19.59 | 45.54 |
| PTK2 | protein tyrosine kinase 2 | 19.44 | 18.64 | 15.26 | 22.26 | 23.03 | 24.01 | 17.78 | 23.10 |
| TLN1 | talin 1 | 244.31 | 160.55 | 101.82 | 219.18 | 212.25 | 271.88 | 168.89 | 234.44 |
